# Pleurisy with pneumothorax due to CAM‐resistant MAC lung disease

**DOI:** 10.1002/rcr2.993

**Published:** 2022-06-14

**Authors:** Yoshihisa Nukui, Tatsuo Kawahara, Satoshi Hanzawa

**Affiliations:** ^1^ Department of Respiratory Medicine Shuuwa General Hospital Saitama Japan

**Keywords:** clarithromycin, drug resistance, *Mycobacterium avium* complex, pleurisy, streptomycin

## Abstract

A 72‐year‐old man received rifampicin, ethambutol and sitafloxacin to treat clarithromycin (CAM)‐resistant *Mycobacterium avium* complex (MAC) lung disease. He was admitted because of fever. Pneumothorax and pleural effusion were present in the right lung, and a new consolidation appeared in the right upper lobe. Based on positive culture of the pleural effusion for CAM‐resistant *M. avium* and findings on chest computed tomography, he was diagnosed with pleurisy due to *M. avium*, with rupture of the subpleural lung parenchymal lesion into the pleural space. Additional treatment with streptomycin (SM) improved the patient's high‐grade fever. SM might be effective for pleurisy caused by CAM‐resistant MAC lung disease.

## Graphical abstract

Here, we describe a case of pleurisy with pneumothorax due to clarithromycin‐resistant *Mycobacterium avium* complex lung disease, which was treated effectively with streptomycin.

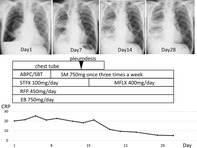

## INTRODUCTION

The prevalence of clarithromycin (CAM)‐resistant *Mycobacterium avium* complex (MAC) lung disease has increased.^1^ Although regimens containing aminoglycoside antibiotics such as streptomycin (SM) and kanamycin have been proposed for the treatment of CAM‐resistant MAC lung disease, treatment of CAM‐resistant MAC lung disease is challenging.^2^ Although non‐tuberculous mycobacteria (NTM) pleurisy is an uncommon manifestation, it has also become a clinical concern with the increasing prevalence of NTM lung disease.^3^ Here, we describe a case of pleurisy with pneumothorax due to CAM‐resistant MAC lung disease, which was treated effectively with SM.

## CASE REPORT

**FIGURE 1:**
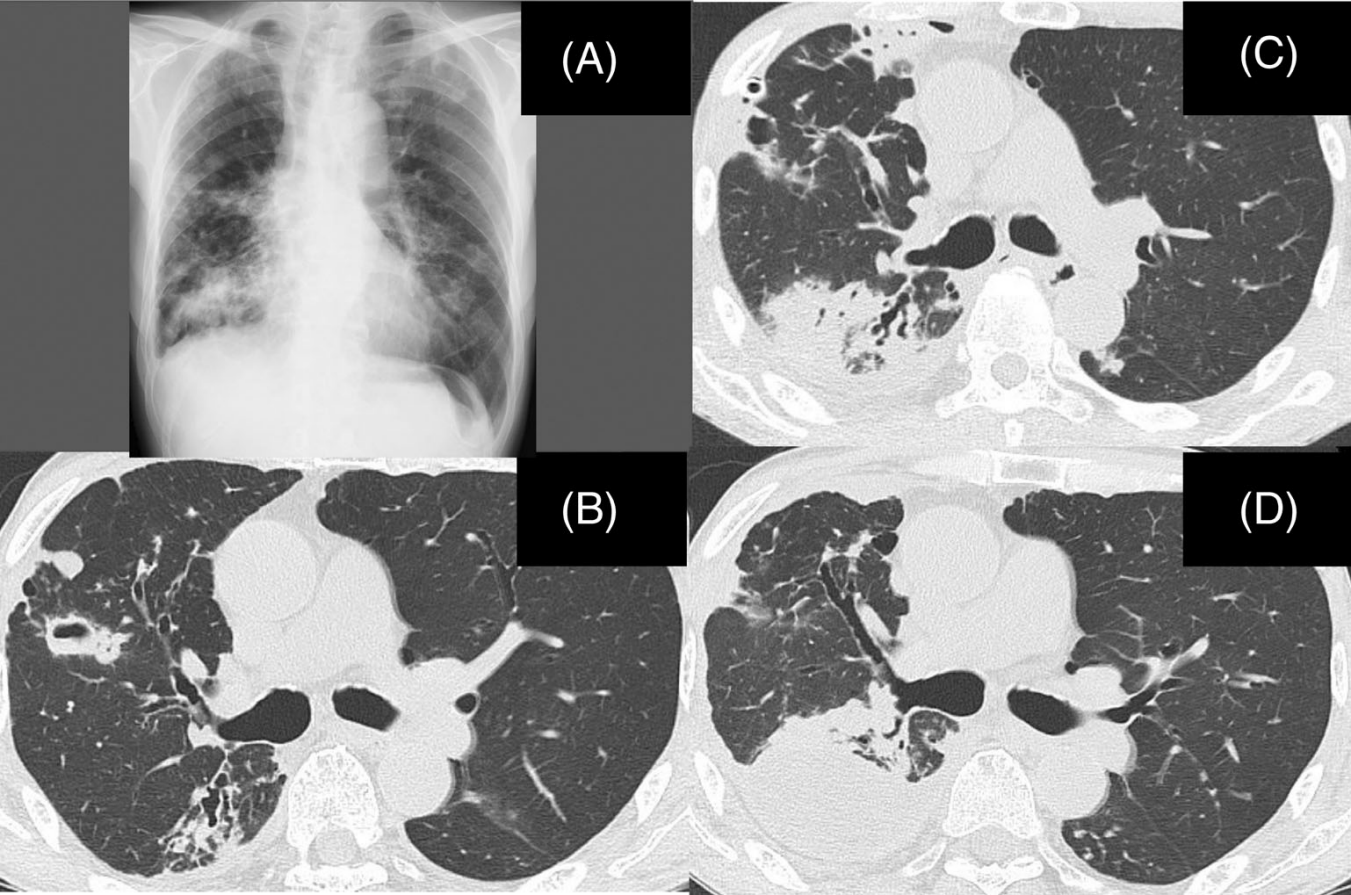
Chest x‐ray revealed consolidation in the right lower lung field and multiple nodules in the bilateral lung fields at 1 year before admission (A). Chest computed tomography (CT) revealed bronchiectasis and multiple small cavities and nodules in both lungs at 1 year before admission (B). Chest CT on Day 7 post‐admission revealed consolidation in the right upper lobe (C). The consolidation in the right upper lobe has decreased, although pleural effusion due to the effects of pleurodesis was observed at 3 months after discharge from hospital (D).

**FIGURE 2:**
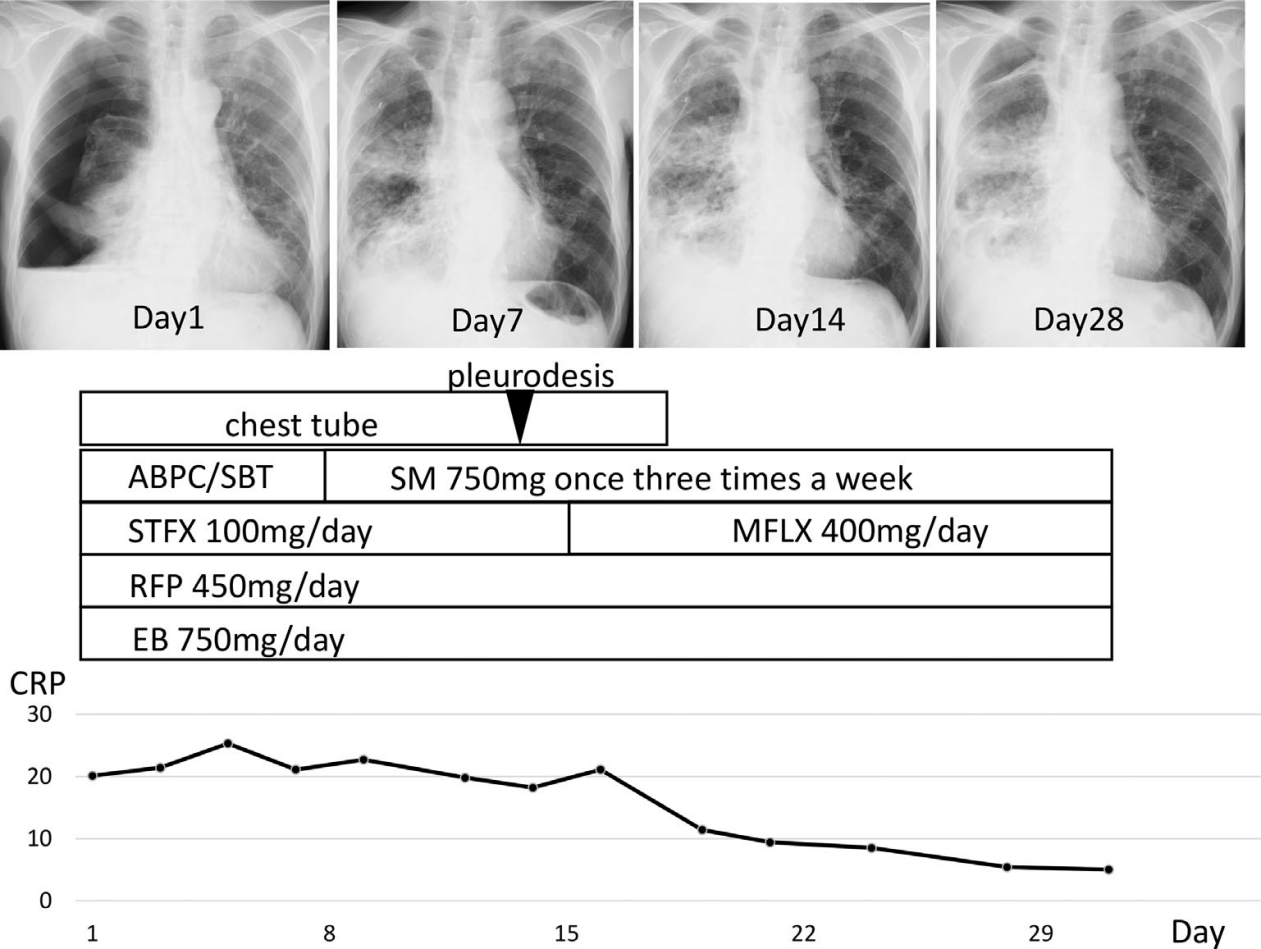
Clinical course of the patients after admission. SM was initiated on the eighth day post‐admission. The CRP level fell gradually to 5.0 mg/dl. ABPC/SBT, sulbactam/ampicillin; CRP, C‐reactive protein; EB, ethambutol; MFLX, moxifloxacin; RFP, rifampicin; SM, streptomycin; STFX, sitafloxacin

A 72‐year‐old man, a former smoker, presented to our hospital with fever. He had a known history of MAC lung disease 16 years earlier, which ultimately led to this visit. He had been treated with rifampicin (RFP), ethambutol (EB) and CAM from 15 years to 5 years before this visit. Because the radiological findings worsened and positive cultures continued, sitafloxacin (STFX) was added to the regimen 5 years before this visit. CAM‐resistant *M. avium* was confirmed 4 years ago, so CAM was withdrawn and aminoglycoside antibiotics were proposed; however, the patient refused therapy due to difficulty in attending the frequent hospital appointments required for intramuscular injection of SM. Subsequently, the radiological findings worsened and positive cultures continued (Figure 1A). On admission, his height, weight, body temperature and the percutaneous oxygen saturation (SpO_2_) level were 163 cm, 48 kg, 38.4°C and 96% on room air, respectively. The white blood cell count was 6670/ml, and C‐reactive protein (CRP) was 20.1 mg/dl The test for HIV was negative. A chest x‐ray on admission revealed pneumothorax and pleural effusion in the right lung. A chest drainage tube was inserted into the right thoracic cavity to treat the pneumothorax. At that time, pleural effusion was collected. Biochemical analysis of the effusion revealed the following: protein, 5.5 g/dl (serum protein, 7.2 g/dl); lactate dehydrogenase (LDH), 2021 IU/L (serum LDH, 141 IU/L); adenosine deaminase, 113.2 U/L; and glucose, 45 mg/dl. Sulbactam/ampicillin (ABPC/SBT) was added soon after admission as the bacteriological results of the effusion were unavailable at that point in time. Despite the treatment, the fever remained unchanged. Then, an effusion smear was negative for acid‐fast bacilli, and cultured isolates were identified as CAM‐resistant *M. avium* (minimal inhibitory concentration > 32 μg/ml). A sputum smear was positive for acid‐fast bacilli, and cultured isolates were identified as CAM‐resistant *M. avium*. No other microorganisms were cultured from sputum and pleural fluid. We diagnosed pleurisy due to *M. avium* on the basis of ineffectiveness of ABPC/SBT and positive culture of the pleural effusion for *M. avium*. On Day 7, pneumothorax improved and consolidation in the right upper lobe was revealed on chest computed tomography (Figure 1A–C). We diagnosed NTM pleuritis due to positive NTM culture of pleural effusion samples.^3^ Although the pathogenesis of NTM pleuritis is uncertain, the rupture of the subpleural focus in the lung into the pleural space could be considered as a trigger in the pathogenesis of this disease.^3^ SM (a single intramuscular injection of 750 mg given three times a week) was initiated on Day 8, and STFX was changed to moxifloxacin (MFLX) on Day 15. The patient's high‐grade fever and CRP level started to improve from around Day 15, suggesting that SM was more effective than MFLX. Pleurodesis with a 50% glucose solution was performed on Day 14. The pneumothorax improved and the chest tube was removed on Day 17. The patient was discharged from hospital on Day 35 (Figure 2). The patient is being treated with a combination of SM, MFLX, EB and RFP; his condition is stable, with a low CRP level (0.6 mg/dl). The consolidation in the right upper lobe has decreased, although pleural effusion due to the effects of pleurodesis was observed at 3 months after discharge from hospital (Figure 1D).

## DISCUSSION

Despite the combination therapy including fluoroquinolone, aminoglycoside and surgical resection, the outcomes of CAM‐resistant MAC lung disease were poor.^4^ Furthermore, some studies have reported worse prognosis of NTM pleuritis.^3^ Our case report suggests that we were able to control the severe state of CAM‐resistant MAC pleuritis with SM.

A previous study has shown that pleural concentrations increase gradually to a maximum value about 6 h after intravenous administration of amikacin.^5^ Moreover, the study also suggested that there might be a significant binding of amikacin to the inflamed pleura. Amikacin is the antibiotic of the same type of SM and belongs to aminoglycoside antibiotics. Based on these references, aminoglycoside antibiotics might be effective for pleural lesions.

## CONFLICT OF INTEREST

None declared.

## ETHICS STATEMENT

The authors declare that appropriate written informed consent was obtained for the publication of this manuscript and accompanying images.

## Data Availability

Data sharing not applicable to this article as no datasets were generated or analysed during the current study.

## References

[1] Prevots DR , Marras TK . Epidemiology of human pulmonary infection with nontuberculous mycobacteria: a review. Clin Chest Med. 2015;36:13–34. 25676516 10.1016/j.ccm.2014.10.002 PMC4332564

[2] Daley CL , Iaccarino JM , Lange C , Cambau E , Wallace RJ Jr , Andrejak C , et al. Treatment of nontuberculous mycobacterial pulmonary disease: an official ATS/ERS/ESCMID/IDSA clinical practice guideline. Eur Respir J. 2020;56:2000535. 32636299 10.1183/13993003.00535-2020 PMC8375621

[3] Yagi K , Ito A , Fujiwara K , Morino E , Hase I , Nakano Y , et al. Clinical features and prognosis of nontuberculous mycobacterial pleuritis, a multicenter retrospective study. Ann Am Thorac Soc. 2021;18:1490–7. 33832404 10.1513/AnnalsATS.202008-938OC

[4] Park Y , Lee EH , Jung I , Park G , Kang YA . Clinical characteristics and treatment outcomes of patients with macrolide‐resistant *Mycobacterium avium* complex pulmonary disease: a systematic review and meta‐analysis. Respir Res. 2019;20:286. 31852452 10.1186/s12931-019-1258-9 PMC6921583

[5] Sakchai L , Pradit C , Chaivej N , Visit U , Yachai NS . Amikacin pharmacokinetics in plasma and pleural fluid. J Med Assoc Thai. 1989;72:90–6. 2500495

